# Willingness of dentists in the United Arab Emirates to perform restorative and surgical treatments for patients infected with hepatitis C

**DOI:** 10.1186/s13690-021-00756-4

**Published:** 2021-12-22

**Authors:** Suhail H Al-Amad

**Affiliations:** College of Dental Medicine, University of Sharjah, Sharjah, United Arab Emirates

**Keywords:** Dentists, Hepatitis C, Attitudes, Ethics

## Abstract

**Background:**

Dentists’ refusal to treat patients infected with hepatitis C (HCV) continues to raise ethical concerns, particularly in countries where HCV is prevalent. The aim of this cross-sectional study was to assess dentists’ willingness to treat patients infected with HCV and the socio-demographic variables that influence their decision.

**Methods:**

An online questionnaire was disseminated to dentists practicing in the United Arab Emirates (UAE) and enquired about their willingness to perform two dental treatments: restorative and surgical, to patients infected with HCV, while hypothetically being equipped with optimal personal protective equipment. Binary logistic regression test was used to assess socio-demographic factors that predict dentists’ unwillingness decision.

**Results:**

Two-hundred and forty-five dentists participated in this survey. Among those, 25.6 and 19.3% were unwilling to perform dental extractions and aerosol-generating restorative dental treatments for patients infected with HCV, respectively. Dentists’ clinical experience was a significant predictor of their unwillingness decision, with those of shorter clinical experience expressing greater reluctance than their counterparts (OR:1.61; 95% CI: 1.02–2.54; *p* = 0.042).

**Conclusion:**

Patients infected with HCV who need dental care could face rejection by early career dentists, particularly if that treatment is a surgical one. Fresh dental graduates should be made aware of their ethical and legal responsibilities towards patients with infectious diseases, particularly HCV.

**Supplementary Information:**

The online version contains supplementary material available at 10.1186/s13690-021-00756-4.

## Background

Globally, there are 180 million persons infected with Hepatitis C virus (HCV) [1]. Contrary to the decreasing prevalence of hepatitis B [2–4] and HIV [5–7], the prevalence of HCV has been increasing, particularly in the Middle East and North Africa (MENA) region, where the age-standardised prevalence exceeds 3.5% [8]. Prevalence of HCV in the United Arab Emirates (UAE) shows a marked variation between the two major societal groups. While the prevalence of HCV has been reported as 0.24% among UAE nationals, the same prevalence surges to 1.64% among expatriates of various nationalities, with the higher rates reported among specific expatriates groups from certain high prevalence countries [9].

Variation in global prevalence of HCV has been attributed to a number of factors, among which was suboptimal cross infection control measures during medical and dental procedures [10, 11]. Detection of HCV in human saliva [12] and on oral mucosal surfaces [13], and its ability to remain viable on various surfaces for weeks [14] make this virus of a particular occupational hazard to dentists and their patients. Associations between HCV occurrence and history of receiving dental treatments have been reported in a number of seroprevalence studies [15–18], with dental treatments having odds ratios ranging from 4.1 to 6.8 [15–18]. As a result, many dentists have expressed their unwillingness to treat patients with HCV out of concerns over their own health [19–21].

Most of the surveys that assessed attitudes of dentists towards blood-borne infections focused on attitudes towards patients with human immune-deficiency and hepatitis B viruses [19, 22–28]. Dentists’ willingness to treat HCV patients has not been sufficiently studied, particularly in the MENA region where HCV prevalence is among the highest globally.

The UAE is an Arabian Gulf state with a prominent multi-national expatriate composition belonging to more than 200 nationalities, some of whom are expatriates from countries with high HCV prevalence, such as Pakistan and Egypt [29]. This country’s unique multinational societal composition poses the question whether the UAE patients’ multinational backgrounds could influence dentists’ willingness to perform dental treatments to patients infected with HCV, when those patients are identified to their dentists after completing the mandatory medical history questionnaire [30].

Previous surveys that assessed dentists’ willingness to treat patients infected with HCV did so using direct questions about the provision of dental treatment in its generic sense [19–21]. It is not known whether dentists can be selective as to the nature of treatment they would be willing to provide to patients infected with HCV.

This research aimed at assessing the willingness of dentists practicing in the UAE to perform two sets of dental treatment to patients infected with HCV: Dental extractions and aerosol-generating restorative dental treatments, while being equipped with optimal PPE.

## Materials and methods

### Design and sample

This survey was part of a larger cross-sectional study that assessed dentists’ dependency on social media for information on infectious diseases [31]. A hyperlink to a Google form was disseminated by Emails, WhatsApp, and Facebook platforms to a convenient sample of dental professionals (dentists and dental specialists) who were actively engaged in providing dental services regardless of their own nationality or cultural and educational backgrounds. First recipients were asked to forward the survey hyperlink to their dental colleagues. Eventually, dentists who indicated that they were practicing in the UAE were selected as the study sample for this study.

### Research tool

**Fig. 1:**
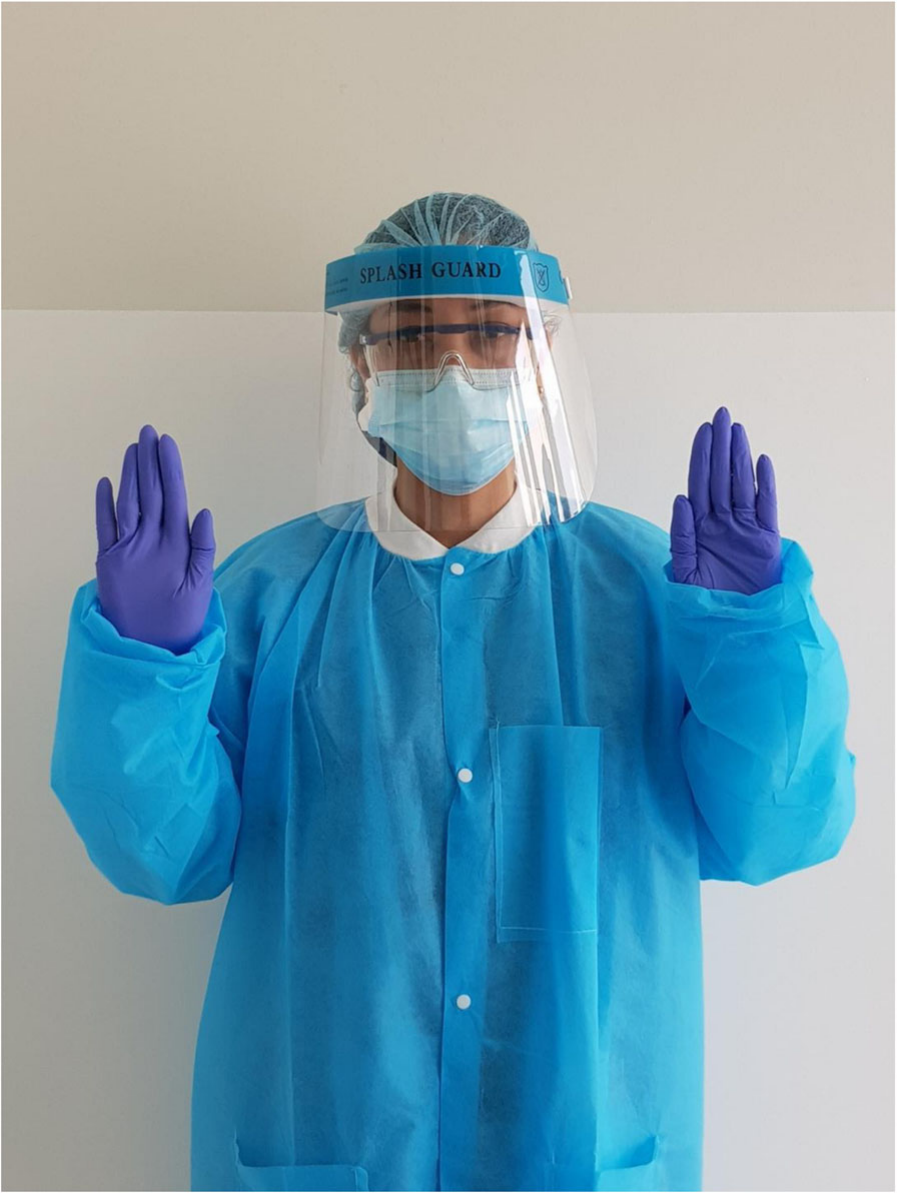
A clinical image that was embedded in the questionnaire showing optimal PPE that participants would hypothetically be equipped with while performing the two sets of dental treatments to patients infected with HCV

The online questionnaire asked about basic socio-demographic variables and the dentists’ willingness to perform two sets of dental treatments: Dental extractions and aerosol-generating restorative dental procedures, to patients who were positive for hepatitis C. In order to eliminate the unavailability or inadequacy of PPE as a factor that might influence dentists’ willingness decision, the questionnaire included an image of a clinician wearing full PPE that participating dentists will hypothetically be equipped with while performing those treatments (Fig. 1).

### Statistical analysis

IBM® SPSS® Statistics (version 27) (IBM Corp. Released 2020. IBM SPSS Statistics for Macintosh, Version 27.0. Armonk, NY: IBM Corp) was used for statistical analysis. Demographic variables were summarized in frequencies and percentages. Chi-square test was used to assess the associations between demographic variables and willingness to treat patients infected with HCV in both treatment sets. Independent samples *t*-test was used to assess differences in the mean values of clinical experience between those willing and unwilling to treat patients infected with HCV. Cohen’s kappa coefficient was used to assess the agreement between dentists’ decision with regards to both sets of treatments. Binary Logistic Regression model was used to identify predictors of unwillingness to treat patients infected with HCV. *p*-value was considered significant if < 0.05.

### Ethical clearance

This research was conducted in accordance with the ethical standards of the 1964 Declaration of Helsinki and its later amendments, and was independently reviewed and approved by an institutional Research Ethics Committee.

## Results

Two-hundred and forty-five (245) dentists participated in this online survey. The mean age of participants was 35.4 years (SD = 9.9), ranging from 23 to 75 years. Females represented 68.4% of the sample. The mean duration of clinical experience was 11.5 years (SD = 9.5), ranging from 1 to 50 years. Two-thirds of the sample were general dentists (*n* = 158 (64.5%)), the remaining were specialists practicing in various dental specialties. Nearly half of the sample were working in the private sector (*n* = 110 (45.3%)), while the other half were working in the public sector (i.e. government dental services or university teaching institutes) (Table 1).

**Table 1:** Description of study sample by socio-demographics characteristics

Variable		N (%)
Experience	< 5 years	91 (37.1)
	5–15 years	81 (33.1)
	> 15 years	73 (29.8)
Sex	Female	167 (68.4)
	Male	77 (31.6)
Sector	Private	110 (45.3)
	Public^a^	133 (54.7)
Education	Undergraduate	125 (51.2)
	Postgraduate	119 (48.8)
Profession category	Dental practitioner	158 (64.5)
	Dental specialist	87 (35.5)

^a^Includes university sector

Despite being equipped with optimal PPE, 25.6 and 19.3% of the surveyed dentists were unwilling to perform dental extractions and aerosol-generating restorative dental treatments for patients infected with HCV, respectively. Cohen’s kappa coefficient showed significant agreement between dentists’ decision with regards to both sets of treatments (κ = 0.59; *p* < 0.000).

Dentists who were willing to perform dental extractions for patients infected with HCV had -on average- 3.3 more years of clinical experience by comparison to those who were unwilling (12.2 years (SD = 9.8) and 8.9 years (SD = 8.3), respectively; *p* = 0.016). No difference was seen for the dentists’ willingness to provide aerosol-generating restorative dental treatments (11.6 years (SD = 9.4) and 10.5 years (SD = 10.1), respectively; *p* = 0.477).

For dental extractions, the willingness decision was significantly associated with shorter clinical experience (*p* = 0.032). No statistically significant association was seen between socio-demographic variables and the dentists’ willingness to perform aerosol-generating restorative dental treatments (*p* = 0.680) (Table 2).

**Table 2:** Bivariate analysis showing the association between dentists’ unwillingness decision towards treating patients infected with HCV and socio-demographic variables

Variable		Performing dental extraction			Performing aerosol-generating treatment		
		Unwilling to treat N (%)	Willing to treat N (%)	*P*-value*	Unwilling to treat N (%)	Willing to treat N (%)	*P*-value*
Total		62 (25.6)	180 (74.4)		47 (19.3)	197 (80.7)	
Experience	1–5	29 (31.9)	62 (68.1)	0.032	20 (22.0)	71 (78.0)	0.680
	6–15	23 (28.4)	58 (71.6)		15 (18.5)	66 (81.5)	
	> 15	10 (14.3)	60 (85.7)		12 (16.7)	60 (83.3)	
Sex	Males	14 (18.4)	62 (81.6)	0.078	11 (14.3)	66 (85.7)	0.174
	Females	48 (29.1)	117 (70.9)		36 (21.7)	130 (78.3)	
Work sector	Private	21 (19.4)	87 (80.6)	0.055	20 (18.2)	90 (81.8)	0.656
	Public	40 (30.3)	92 (69.7)		27 (20.5)	105 (79.5)	
Education	Undergraduate	35 (28.0)	90 (72.0)	0.402	27 (21.6)	98 (78.4)	0.359
	Postgraduate	27 (23.3)	89 (76.7)		20 (16.9)	98 (83.1)	
Profession category	Dentist	42 (26.6)	116 (73.4)	0.638	31 (19.6)	127 (80.4)	0.848
	Specialist	20 (23.8)	64 (76.2)		16 (18.6)	70 (81.4)	

*Based on Chi-square test

Binary Logistic Regression showed that dentists’ shorter clinical experience was a significant predictor of their willingness to treat patients infected with HCV (OR: 1.61, 95% CI: 1.02–2.54; *p* = 0.042). This prediction was independent from the dentists’ sex, work sector, education, and professional category (Table 3).

**Table 3:** Binary Logistic Regression model identifying predictors of the unwillingness to perform dental extraction to patients infected with HCV

Variable	β	S.E.	Adjusted OR (Exp β)	95% confidence interval		*P*-value
				Lower	Upper	
Experience	0.475	0.233	1.609	1.018	2.542	0.042
Sex (female, male^a^)	−0.446	0.378	0.64	0.305	1.343	0.238
Sector (private, public^a^)	−0.459	0.329	0.632	0.332	1.204	0.163
Education (undergraduate, postgraduate^a^)	−0.141	0.504	0.868	0.323	2.331	0.779
Professional category (dentist, specialist^a^)	−0.13	0.539	0.878	0.305	2.523	0.809

^a^Reference category

## Discussion

Several seroprevalence studies reported associations between the occurrence of HCV and history of receiving dental treatments, making HCV a significant occupational hazard of dental practice. As a result, many dentists from various countries have expressed reluctance to provide dental treatments to patients infected with HCV out of fear of themselves becoming infected with this virus [19–21].

In this cross-sectional study, dentists’ willingness to treat patients infected with HCV was assessed against two forms of dental treatments, each with a different infectivity hazard namely: dental extractions and aerosol-generating dental procedures. This design allowed investigating whether dentists’ reluctance to treat those patients was an absolute one, or could be influenced by the nature of dental treatment being provided (i.e. surgical or restorative).

Results of this study showed that dentists were selective with regards to the dental treatment they would be willing to provide to patients infected with HCV, with surgical treatments having higher rates of reluctance by comparison to restorative ones (26 and 19%, respectively).

Unwillingness rates reported here fall within the range reported elsewhere in which 15–68% of dentists were unwilling to treat patients with blood-borne infections, namely Hepatitis B and HIV [19, 22–28]. Traditionally, unwillingness rates have been associated with various socio-demographic factors, such as the dentists’ age, sex, and length of clinical experience, with most studies reporting greater unwillingness attitudes among female dentists with shorter clinical experiences [21, 24, 28].

In our study, dentists who were unwilling to treat patients infected with HCV were almost 3 years younger in experience than those who were willing to treat those patients. A logistic regression model strengthened this finding by showing that shorter clinical experience was a single predictor of the dentists’ unwillingness decision, adjusted for sex, work sector, educational level and professional status (generalists or specialists).

Refusal to treat patients with infectious diseases because of healthcare providers’ concerns over their own safety has raised legal and ethical questions [32–34]. Healthcare providers’ refusal to provide their patients with specific treatments (such as abortion) has commonly been viewed as ethically and legally admissible, when this refusal contradicts the providers’ cultural or religious beliefs [35]. During the COVID-19 global pandemic, a number of authors opined that healthcare providers’ refusal to treat patients with infectious diseases should be made admissible, when their refusal is justified by unavailability or inadequacy of PPE [36–39].

In order to eliminate unavailability or inadequacy of PPE as factors that might influence dentists’ decision with regards to treating patients with HCV, our questionnaire included an image of a clinician wearing optimal PPE that dentists would hypothetically be equipped with while performing the said dental treatments (Fig. 1). The results reported here -therefore- represent a better reflection of the dentists’ attitudinal decisions, without PPE insufficiency being an influencing factor.

This study revealed a number of important findings. Firstly, dentists’ unwillingness to treat patients infected with HCV is not an absolute one but is influenced by the hazardous nature of specific dental treatments. Secondly, the fact that 1 in 4 dentists were unwilling to perform dental extractions to patients infected with HCV, despite being fully equipped with optimal PPE, infers a lack of confidence in the protective efficacy of those equipment, a professional stance that can be detrimental to all patients with infectious diseases. Finally, the high rate of dentists, particularly early career ones, who are unwilling to treat patients infected with HCV should draw attention towards the professional preparedness of fresh dental graduates with regards to their ethical and legal responsibilities towards their patients.

Several papers have addressed poor knowledge among dental students towards blood-borne infectious diseases, including HCV [19, 40, 41], calling for strengthening dental curricula in the fields of infectious diseases and cross infection control in the clinical setting. This study adds to previous calls for enhancing the knowledge of dental students in the biological hazards of dental practice, as well as their confidence in the protective efficacy of PPE in all forms of treatments, including surgical ones.

Despite those outcomes, our study is limited by the sample selection that was based on convenience, and by using the general definition of HCV positivity without specifying if that positivity was related to the presence of the active virus or the presence of serum antibodies. The hypothetical design of this research, in which adequacy of PPE and nature of dental treatment have been pre-determined for participating dentists, was specifically intended to create a clinical scenario that is as much approximate as possible to a real-life setting. Other variables, such as cultural and religious beliefs, financial revenues, and governing laws, can influence dentists’ decision but have not been investigated here. Direct observational studies based on mystery shoppers [28] are needed to accurately assess professional attitudes towards providing dental treatments of various biological hazard to patients with blood-borne infectious diseases.

## Conclusion

Despite being equipped with optimal PPE, 1 in 4 dentists practicing in the UAE were unwilling to treat patients infected with HCV. Young dentists should be made aware of their ethical and legal responsibilities towards patients with infectious diseases, and to the protective efficacy of PPE in preventing the transmission of infectious diseases from patients to care providers.

## Supplementary Information

**Additional file 1.**

## Data Availability

The datasets during and/or analysed during the current study available from the corresponding author on reasonable request.

## Abbreviations

HCV: Hepatitis C virus
HIV: Human immune-deficiency virus
MENA: Middle East and North Africa
PPE: Personal protective equipment
UAE: United Arab Emirates
