# Supplementary material for: Willingness of dentists in the United Arab Emirates to perform restorative and surgical treatments for patients infected with hepatitis C
Source: Arch Public Health. 2021 Dec 22;79:230. doi: 10.1186/s13690-021-00756-4 (PMC8692077; doi:10.1186/s13690-021-00756-4)
Supplement: Supplementary file 1 — Additional file 1. [file 13690_2021_756_MOESM1_ESM.docx]

**Section A: Demographic data**

**Tell us a little bit about yourself**

1. What is your age?

________

1. How many years have you been practicing dentistry?

_______

1. What is your sex?

- Male
- Female

1. What your highest qualification?

- Diploma
- Bachelor
- Higher Diploma
- Master
- PhD
- Clinical doctorate
- Professional specialization degrees, such as fellowships and board certificates

1. Are you a

- Dentist
- Dental specialist
- Dental hygienist
- Dental nurse
- Dental technician
- Dental intern

1. Are you working in a

- Private clinic
- Government clinic
- University teaching clinic
- Others

1. Which country are you currently working in?

______

1. If you are a dental specialist, what is your specialty

- Periodontics
- Prosthodontics
- Restorative/conservative dentistry
- Endodontics
- Oral surgery, or oral and maxillofacial surgery
- Orthodontics
- Paediatric dentistry
- Oral medicine, or oral pathology, or oral radiology
- Others

**Section B: Your infection control measures**


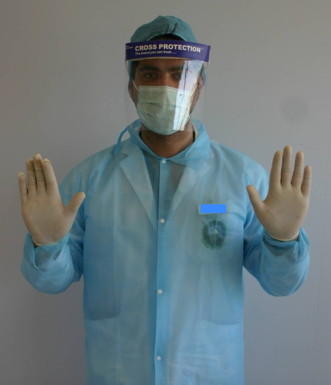


Using the standard PPE (as seen in the photograph below), how comfortable are you performing the following procedures, or assisting a dentist while performing them?

1. How comfortable are you performing an AEROSOL-GENERATING dental treatment for the following patients, while wearing the PPE showing in this image?

|  | Not comfortable at all | Little comfortable | Somewhat comfortable | Very comfortable |
| --- | --- | --- | --- | --- |
| A confirmed case of hepatitis C |  |  |  |  |

1. How comfortable are you performing a NON-SURGICAL DENTAL EXTRACTION for the following patients, while wearing the PPE showing in this image?

|  | Not comfortable at all | Little comfortable | Somewhat comfortable | Very comfortable |
| --- | --- | --- | --- | --- |
| A confirmed case of hepatitis C |  |  |  |  |

Thank you for sparing time to participate in our survey
